# Symptom Management for Patients With Esophageal Cancer After Esophagectomy

**Published:** 2016-11-01

**Authors:** Laura A. Pachella, Susan Knippel

**Affiliations:** From the Department of Thoracic Surgery, The University of Texas MD Anderson Cancer Center, Houston, Texas

## Abstract

**CASE STUDY**

KD is a 67-year-old man with a medical history of hypertension, asthma, and a 20-pack/year smoking history who developed progressive dysphagia 8 months ago. Upon consultation with his primary care provider, he underwent an esophagogastroduodenoscopy (EGD) for evaluation. A friable mass was visualized at the gastroesophageal junction, and biopsies confirmed adenocarcinoma of the esophagus. KD completed a staging evaluation with positron-emission tomography/computed tomography (PET/CT), which did not reveal distant metastatic disease. He also had an endoscopic ultrasound (EUS), which showed the tumor invading the muscularis propria and did not identify any enlarged regional lymph nodes (stage T3N0 disease).

KD was referred to a medical oncologist and a radiation oncologist; he underwent concurrent chemoradiation therapy with docetaxel and fluorouracil and radiation therapy (50.4 Gy). KD was referred to thoracic surgery following restaging with PET/CT and EGD; there was no evidence of distant metastatic disease, and pathology findings revealed residual adenocarcinoma in one of the four esophageal biopsies.

KD underwent Ivor Lewis esophagectomy and had a jejunostomy tube placed for nutritional requirements for 10 weeks as he adjusted to oral nutrition. Surgical pathology findings revealed residual adenocarcinoma with treatment effect; no malignancy was detected in the sampled regional lymph nodes.

Four months later, KD presents with complaints of frequent postprandial diarrhea and reflux. He says he has been trying to lie down after meals due to palpitations and flushing. He is anxious about these symptoms and fearful about his long-term prognosis adjusting to the side effects of esophagectomy and would like to discuss lifestyle modifications.

It was estimated that 16,980 new cases of esophageal cancer were diagnosed in the United States in 2015, and 15,590 patients died of the disease (Surveillance, Epidemiology, and End Results [SEER], 2015). Esophageal cancer is a small percentage of the total malignancies diagnosed in the United States; however, its incidence has been rising.

Squamous cell carcinoma has typically been the more prevalent form of the disease and is believed to be due to chronic irritation to the esophagus from excessive alcohol consumption and smoking (Zhang, 2013). Adenocarcinoma has become the more common form of the disease in the United States, as it has increased rapidly since the 1970s. The exact cause remains unclear; however, smoking, obesity, gastroesophageal reflux disease, and *Helicobacter pylori* infection have been studied (Thrift & Whiteman, 2012).

Oncology advanced practitioners (APs) are called upon to have expertise in caring for this subset of patients. Esophageal cancer has been shown to be most effectively treated with a trimodality approach, including chemotherapy, radiation therapy, and surgical resection with esophagectomy (National Comprehensive Cancer Network [NCCN], 2015). Esophagectomy is performed by several techniques and involves resection of the esophagus, dissection of regional lymph nodes, and creation of a conduit (Lin, Akhter, & Iannettoni, 2005, p 2,285).

There have been several reports on quality-of-life changes surrounding patients after esophagectomy, although little has been published on the practical management of symptoms in a comprehensive article. The physical symptoms and psychosocial issues related to esophageal cancer are life-altering in regard to the patient’s overall health and social interactions. Possible symptoms unique to patients after esophagectomy include anastomotic stricture, dumping syndrome, reflux, and gastroparesis (Ginex et al., 2013). The severity of symptoms may change over time due to alleviation through lifestyle modifications, or the patient’s perception may change, as many of the symptoms are self-reported and difficult for clinicians to measure (Darling, 2013).

Esophagectomy changes a patient’s quality of life, and this topic has been widely studied and reported in the medical and nursing literature. However, there is a scarcity of information on the management of symptomatology of patients with esophageal cancer after esophagectomy. The purpose of this review is to offer an overview of common side effects of esophagectomy and interventions to minimize the symptoms.

## ESOPHAGEAL STRICTURE

Esophageal anastomotic stricture is one of the most common complications after esophagectomy, occurring in 10% to 40% of all patients; symptoms include dysphagia and postprandial fullness (Huang et al., 2015). Up to one-third of patients who undergo an Ivor Lewis approach develop anastomotic stricture (Nafteux et al., 2009, p 160).

Esophageal stricture typically develops 2 to 6 months after surgery and may be due to tension and an inadequate blood supply (Engstad & Schipper, 2009, p 664). Higher rates have been reported in patients who have had an anastomotic leak after surgery (Gamliel & Krasna, 2009, p 139). Anastomotic stricture was found to be independently associated with poor global quality of life (Huang et al., 2015). Patients may relate swallowing problems to recurrence of cancer and may become alarmed at the development of these symptoms (Tsottles & Reedy, 2005, p 1265–1266).

Evaluation for esophageal stricture includes a barium swallow, esophagogastroduodenoscopy (EGD), and computed tomography (CT) of the chest (Argote-Greene & Sugarbaker, 2009, p 151). It is important to note that postoperative dysphagia should be evaluated with endoscopic or radiographic confirmation, as one-third of patients with dysphagia will not have stricture (Engstad & Schipper, 2009, p 664).

**Interventions**

The initial intervention for esophageal stricture is dilation, which involves expansible forces against a luminal stenosis. Bougie dilators or balloon dilators can be used through an endoscopic procedure using a dilator and guidewire (Siddiqui et al., 2013). Between 80% and 90% of patients can be treated successfully with endoscopic dilation; however, some patients may need to undergo dilation every 2 to 3 weeks until there is a complete response (van Boeckel & Siersema, 2015). If a stricture is persistent after dilation, the patient may need an esophageal stent, which would allow for the use of radial expandable forces. The stent is potentially removable if the stricture resolves after some time (Siddiqui et al., 2013).

There has been conflicting evidence on the benefit of steroid injections in patients with anastomotic stricture. Early studies were conducted on patients with stricture related to peptic ulcer disease and showed promise. However, there has been limited clinical benefit in patients for whom stricture is related to anastomotic changes (van Boeckel & Siersema, 2015). Sugimura and colleagues (2016) described in one study that patients who received steroid injection for dilation with anastomotic stricture from esophagectomy had a longer period between dilations and a reduced number of endoscopies for symptomatic relief.

Self-dilation is an option for refractory proximal esophageal anastomotic stricture. There are advantages to self-dilation, as it is an outpatient procedure that the patient performs at home and there is no need for the use of fluoroscopy or EGD; however, the patient would need to be comfortable with the technique for it to be effective (Chang & Orringer, 2007). Zehetner and colleagues (2014) reported on 16 patients who were able to perform home self-dilation for esophageal stricture with safe and effective results.

## DUMPING SYNDROME

Dumping syndrome is a clinical diagnosis distinguished by gastrointestinal symptoms including postprandial cramping, bloating, nausea, diarrhea, and vasomotor symptoms of flushing, diaphoresis, syncope, and palpitations (Engstad & Schipper, 2009, p 663). The mechanisms of dumping syndrome are due to the response from the rapid passage of food through the gastrointestinal tract related to alterations in gastrointestinal paracrine hormone secretion as well as anatomic factors related to surgery and subsequent reconstruction (Berg & McCallum, 2016).

Early dumping syndrome, which is more common, is described as vasomotor and gastrointestinal symptoms that are attributed to rapid gastric emptying or rapid exposure of the small intestine to nutrients (Ukleja, 2005). Late dumping syndrome occurs 1 to 3 hours after eating and is described as reactive hypoglycemia, including perspiration, faintness, decreased concentration, and altered levels of consciousness. It is noted that patients with late dumping symptoms may also experience early dumping symptoms as well (Berg & McCallum, 2016; Didden, Penning, & Masclee, 2006). Patients may decrease their food intake to relieve symptoms; however, this may lead to weight loss and malnutrition (Ukleja, 2005). Diagnosis is typically made by symptoms, although more specific testing can be obtained if the diagnosis is not clear, including a glucose tolerance test.

**Interventions**

There are several interventions to mitigate the symptoms of dumping syndrome. Dietary modification is the most practical and conservative measure through the avoidance of high carbohydrate loads including simple sugars. Altering traditional meal times to move to small frequent meals, about 6 per day, and restricting fluid intake within meals have been shown to help the majority of patients. Fluids with meals can increase gastric motility, so such fluids should be restricted with meals and 30 minutes after eating. Dairy products may need to be avoided. An increase in fiber intake has been shown to treat hypoglycemia, whereas proteins and fats have been shown to decrease symptoms (Berg & McCallum, 2016). Patients need consultation with a registered dietician to be sure the appropriate amount of calories is taken in while minimizing symptoms. Many patients will need to experiment with different types of foods to understand their food preference and tolerance (Ukleja, 2005).

Some patients do not respond to dietary changes and may require pharmacologic therapy. Medication can be used to relieve the vasomotor symptoms of dumping, including propranolol, prednisone, and verapamil. Octreotide has shown to be effective in some patients by delaying gastric emptying and small bowel motility while also inhibiting insulin release (Engstad & Schipper, 2009, p 664; Ukleja, 2005). A longer-acting formulation of octreotide (Sandostatin LAR) has been found to be more convenient for long-term use in patients with severe symptoms, as it does not require daily subcutaneous injections; reduction of dumping syndrome has been reported in about 50% of patients with prolonged use of this agent (Didden et al., 2006). Referral to a gastroenterologist is recommended for patients who have unmanageable symptoms despite conservative measures and may require the use of medication.

## GASTROPARESIS

Gastroparesis is defined as delayed gastric emptying in the absence of mechanical obstruction (Camilleri, Parkman, Shafi, Abell, & Gerson, 2013). In patients who underwent esophagectomy, gastroparesis may result from denervation of the gastric conduit and disruption of the pyloric function secondary to vagotomy (Engstad & Schipper, 2009, p 664). Gastroparesis may be caused early in the postoperative period by edema in the mucosa and generally resolves within 10 to 14 days (Battafarano & Patterson, 2008, p 548). Prolonged clinical manifestations are noted in 10% to 50% of patients after esophagectomy (Argote-Greene & Sugarbaker, 2009, p 153). Symptoms of gastroparesis include nausea, vomiting, early satiety, and epigastric pain. Radiographic confirmation can be obtained through dilation of the gastric conduit on chest x-ray or delay on barium swallow (Akkerman, Haverkamp, Van Hillegersberg, & Ruurda, 2014).

**Interventions**

Treatment of gastroparesis focuses on symptom management, including dietary changes and ensuring that patients follow the postesophagectomy diet (eating small frequent meals as opposed to three large meals). Foods to avoid in patients with gastroparesis include high-fat and high-fiber foods, as they decrease gastric motility (Fogle, 2013). Gastroparesis can be treated with pyloric dilation after surgery. Intraoperative Botox injection has been noted to decrease gastroparesis in the postoperative period (Akkerman et al., 2014). Intraoperative procedures to improve gastric emptying (including pyloric drainage) are routinely preformed; however, there are mixed data as to their effectiveness (Li et al., 2014).

Medical therapy is aimed at treating the underlying symptoms and increasing gastric motility. Metoclopramide is the only drug in the United States approved by the U.S. Food and Drug Administration (FDA) for the indication of gastroparesis, although its use is controversial due to the side effects of sedation, restlessness, and acute dystonia (Stein, Everhart, & Lacy, 2015). Domperidone is available internationally and through special permission from the FDA as a prokinetic agent; however, it is not approved in the United States, as it has been associated with fatal cardiac events. Erythromycin has also been used in the treatment of gastroparesis, as it is a motilin agonist, although tachyphylaxis develops and adverse cardiac events may also be an issue (Stein et al., 2015). Referral to a gastrointestinal specialist is recommended for patients who are refractory to conservative measures.

## REFLUX

Reflux is a common postoperative change in patients who undergo esophagectomy and reconstruction, as the procedure disrupts normal antireflux mechanisms. The disturbance of positive abdominal pressure and negative thoracic pressure promotes reflux (Engstad & Schipper, 2009, p 664). Patients most commonly report pain, cough, and aspiration, and the severity of the symptoms may be related to the site of the anastomosis.

**Interventions**

Symptomatic patients may require the use of medical therapy such as proton pump inhibitors or histamine 2 (H2) receptor blockers (Engstad & Schipper, 2009, p 664). Additional interventions include small, frequent feedings and avoidance of fluids with meals. Elevation of the patient’s head while lying in bed (at least a 30 angle) is critical to the prevention of aspiration pneumonia. Avoiding meals 3 hours before bed should also limit the amount of reflux.

The use of proton pump inhibitors is common in the treatment of reflux, and the side effects have been well studied in recent years, with research ongoing. The potential harm of proton pump inhibitors involves micronutrient absorption, including iron, calcium, magnesium, and vitamin B12, which may lead to a host of problems including osteoporosis. Drug interactions should be noted, particularly with clopidogrel, as proton pump inhibitors and clopidogrel share the same cytochrome P450 pathway, leading to possible decreased efficacy of the platelet aggregation inhibitor in cardiac patients. Enteric infections with *Clostridium difficile* have been linked to suppression of acid production (Corleto, Festa, Di Giulio, & Annibale, 2014).

## DISCUSSION

There are several physical symptoms related to esophagectomy (see Table). The strategy of preventing symptoms, as opposed to alleviating them, is being researched by surgeons, who have studied changes in technique that might decrease side effects from surgery (Akkerman et al., 2014). There has not been shown to be a detectable difference in quality of life in the location of anastomosis (cervical vs intrathoracic), which may be due in part to the patient’s perception and ability to adjust to lifestyle changes (Wormald, Bennett, van Leuven, & Lewis, 2016).

**Table 1:**
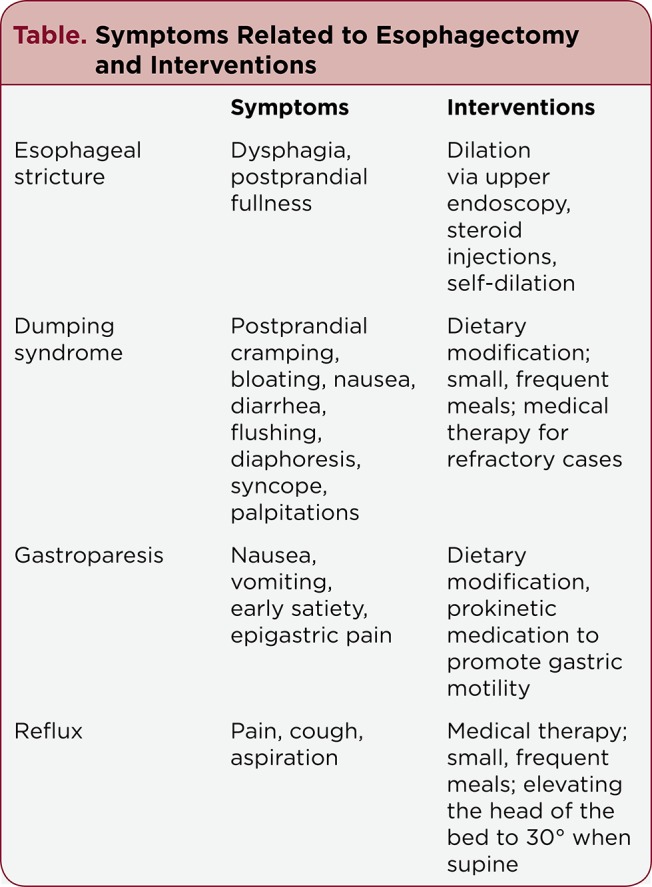
Symptoms Related to Esophagectomy and Interventions

## THE ROLE OF THE ADVANCED PRACTITIONER

The first role of the oncology AP is to educate patients before surgery is offered. It is an important part of patient autonomy that the full scope of lifestyle change is thoroughly discussed. Although hope can be offered through the quality of life research that has been mentioned, patients need to understand the life-changing effects that may happen initially. Patients will need to learn to adjust their eating and sleeping patterns, which will affect their social interactions or possibly employment. It has been described that patients who had more physically demanding jobs were more likely to discontinue working after esophagectomy than were patients with sedentary jobs (Pinto et al., 2016). Connecting potential surgical candidates with patients who previously went through esophagectomy and support groups may promote a better understanding for patients of what surgery entails.

Following surgery, most patients will require an enteral feeding tube while transitioning to a postesophagectomy diet. This also directly impacts patients’ ability to return to a regular routine, as those who are more dependent on jejunostomy feedings are noted to have a delayed return to work (Pinto et al., 2016). Advanced practitioners should follow patients in the postoperative recovery period to monitor progress on nutrition and adjustment to changes in digestion. Registered dieticians play a pivotal role, ensuring that patients are taking in enough calories while adjusting to the new diet. Screening for symptoms related to surgical changes of esophagectomy is a long-term issue for oncology APs.

The results of multiple quality-of-life studies showed that patients who undergo esophagectomy for esophageal cancer report poor outcomes initially after surgery. The vast majority of patients will return to their preoperative quality of life within 6 months to 1 year (Barbour et al., 2008; Darling, 2013; Ginex et al., 2013; Huang et al., 2015; Ramakrishnaiah, Dash, Pal, Sahni, & Kanti, 2014; Shen, Wang, Li, Yi, & Wang, 2015). Advanced practitioners need to prepare patients undergoing esophagectomy for the potential prolonged recovery and adjustment in lifestyle. Patients can be offered hope that within a year, it may be possible for them to return to a baseline of health. Advanced practitioners can serve as a resource as patients adjust to symptoms and recognize severe symptoms that may require additional intervention.

## CONCLUSION

Esophagectomy is a life-changing procedure that is known to contribute to symptoms that will impact a patient’s quality of life. Patients with esophageal cancer and their caregivers look to their oncology providers as experts in this disease, which makes up only 1% of all cancers in the United States. Oncology APs who work with patients after esophagectomy need specialized skills and knowledge to care for these patients. Additional research is needed on the side effects of esophagectomy and other related treatments in esophageal cancer.
